# Glutamine-Derived Aspartate Biosynthesis in Cancer Cells: Role of Mitochondrial Transporters and New Therapeutic Perspectives

**DOI:** 10.3390/cancers14010245

**Published:** 2022-01-04

**Authors:** Ruggiero Gorgoglione, Valeria Impedovo, Christopher L. Riley, Deborah Fratantonio, Stefano Tiziani, Luigi Palmieri, Vincenza Dolce, Giuseppe Fiermonte

**Affiliations:** 1 Department of Bioscience, Biotechnology and Biopharmaceutics, University of Bari, 70125 Bari, Italy; rugorgo@gmail.com (R.G.); valeriaimpedovo@utexas.edu (V.I.); fratantoniod@gmail.com (D.F.); luigi.palmieri@uniba.it (L.P.); 2 Department of Molecular Biosciences, College of Natural Sciences, The University of Texas at Austin, Austin, TX 78712, USA; chrisloganriley@gmail.com; 3 Department of Nutritional Sciences, The University of Texas at Austin, Austin, TX 78712, USA; tiziani@utexas.edu; 4 Department of Pediatrics, Dell Medical School, The University of Texas at Austin, Austin, TX 78723, USA; 5 Department of Oncology, Dell Medical School, LiveSTRONG Cancer Institutes, The University of Texas at Austin, Austin, TX 78723, USA; 6 Department of Pharmacy, Health and Nutritional Sciences, University of Calabria, 87036 Rende, Italy

**Keywords:** cancer, glutamine metabolism, aspartate, mitochondrial carriers, UCP2, SLC1A5_var, aspartate/glutamate carrier, glutamate carrier

## Abstract

**Simple Summary:**

In recent years, aspartate has been increasingly acknowledged as a critical player in the metabolism of cancer cells which use this metabolite for nucleotide and protein synthesis and for redox homeostasis. Most intracellular aspartate derives from the mitochondrial catabolism of glutamine. To date at least four mitochondrial transporters have been involved in this metabolic pathway. Their involvement appears to be cancer type-specific and dependent on glutamine availability. Targeting these mitochondrial transporters may represent a new attractive strategy to fight cancer. The aim of this review is to dissect the role of each of these transporters in relation to the type of cancer and the availability of nutrients in the tumoral microenvironment.

**Abstract:**

Aspartate has a central role in cancer cell metabolism. Aspartate cytosolic availability is crucial for protein and nucleotide biosynthesis as well as for redox homeostasis. Since tumor cells display poor aspartate uptake from the external environment, most of the cellular pool of aspartate derives from mitochondrial catabolism of glutamine. At least four transporters are involved in this metabolic pathway: the glutamine (SLC1A5_var), the aspartate/glutamate (AGC), the aspartate/phosphate (uncoupling protein 2, UCP2), and the glutamate (GC) carriers, the last three belonging to the mitochondrial carrier family (MCF). The loss of one of these transporters causes a paucity of cytosolic aspartate and an arrest of cell proliferation in many different cancer types. The aim of this review is to clarify why different cancers have varying dependencies on metabolite transporters to support cytosolic glutamine-derived aspartate availability. Dissecting the precise metabolic routes that glutamine undergoes in specific tumor types is of upmost importance as it promises to unveil the best metabolic target for therapeutic intervention.

## 1. Introduction

For many years, following Otto Warburg’s pioneering work [1], biologists have focused their attention on glucose metabolism in cancer cells. The interest in the tight connection between tumor growth and glucose utilization further increased with the later findings that genes, usually mutated in human cancers, boosted glucose metabolism [2,3,4]. Although cancer cells convert most of the glycolytic pyruvate to lactate, even in the presence of oxygen [1,4,5], their survival and proliferation rely on mitochondrial activity to provide the building blocks for macromolecule synthesis [6]. One of the most important nutrients used by cancer cells to fuel anabolic processes is glutamine [7]. Glutamine, a source of carbon and reduced nitrogen for many biosynthetic reactions, has an anaplerotic function in cancer cells and controls redox homeostasis [6,8,9,10,11]. To support these cellular needs, glutamine enters several metabolic pathways in both the cytosol and the mitochondria. In this review, we will focus on the role of mitochondrial glutamine metabolism. For an overview of the cytosolic metabolism of glutamine the reader is refered to several excellent reviews [9,12,13].

The two main metabolites derived from mitochondrial glutamine oxidation, when the Krebs cycle (KC) is fully functioning [14,15], are malate and aspartate. Both are produced in the matrix and used in the cytosol to produce NADPH for anabolic processes and redox homeostasis. Aspartate, specifically, is crucial for protein and nucleotide biosynthesis [11,16,17]. Importantly, it was recently shown that the main purpose of the mitochondrial electron transport chain (ETC) in cancer cells’ proliferation is to enable mitochondrial aspartate synthesis [18,19,20]. Inhibitors of the ETC and hypoxic conditions negatively affect the proliferation of many cancer cells by reducing the availability of aspartate [21].

**Figure 1:**
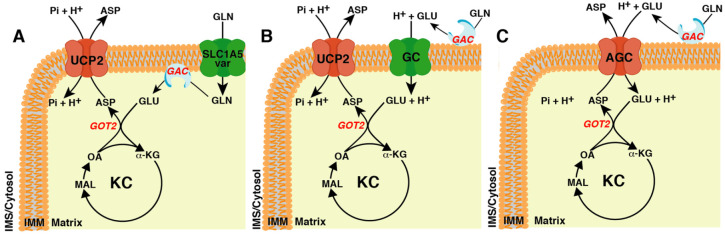
The different transporters involved in cytosolic glutamine-derived aspartate availability based on submitochondrial localization of glutaminase. (**A**) The localisation of glutaminase (GAC) at the matrix side of the inner mitochondrial membrane (IMM) requires the glutamine transporter and the aspartate/Pi + H^+^ exchanger (uncoupling protein 2, UCP2). (**B**) The localisation of GAC at the external side of the IMM requires the glutamate + H^+^ transporter (GC) and UCP2. (**C**) The localisation of GAC at the external side of the IMM requires only the aspartate/glutamate + H^+^ carrier (AGC). ASP, aspartate; GLN, glutamine; GLU, glutamate; Pi, phosphate; OA, oxaloacetate; MAL, malate; α-KG, α-ketoglutarate; KC, Krebs cycle; IMS, intermembrane space; GOT2, mitochondrial isoform of the glutamate-oxaloacetate transaminase.

In order to produce aspartate, glutamine must enter the KC as α-ketoglutarate (α-KG). The critical enzyme in this metabolic pathway is glutaminase (GA) [22]. This enzyme converts glutamine to glutamate, which is further transformed by glutamate dehydrogenase (GDH) or glutamate-oxaloacetate transaminase (GOT) to α-KG. At least four isoforms of GA [23], with a different subcellular localization (mitochondrial, cytosolic, and nuclear) [24,25,26], are present in humans. Here, we will focus on glutaminase C (GAC or GLS C) [23], a shorter isoform of the kidney type GA (KGA or GLS) that is localized to mitochondria [26] and frequently induced in cancer cells [23]. Although it is commonly accepted that GAC is bound to the inner mitochondrial membrane, the localisation of its catalytic site, whether in the matrix or in the intermembrane space, is still a matter of debate [26,27,28,29,30]. This is a crucial point because if the catalytic site is matrix facing a glutamine transporter would be required (Figure 1A). However, if the catalytic domain faces the intermembrane space, glutamate would be released outside the mitochondria thus requiring a glutamate transporter [31,32,33].

The latter hypothesis poses another question: does glutamate enter into mitochondria through GC (Figure 1B) or AGC (Figure 1C)? Although the two routes would be equally efficient as far as glutamate import is concerned, they are different with respect to aspartate export. Indeed, if glutamate enters into the mitochondria through AGC, the aspartate produced in the matrix can exit through this transporter in exchange for glutamate (Figure 1C), whereas if glutamate enters through GC, an aspartate exporter is needed, e.g., UCP2 (Figure 1B).

As discussed below, data reported in the literature support all of these scenarios in different contexts suggesting the route of transport depends on the cancer type and glutamine availability in the tumor microenvironment.

## 2. Glutaminase: A Multifaceted Enzyme in Cancer Cell Metabolism

Before beginning with the role of the mitochondrial carriers in the aspartate metabolism of cancer cells, it is worth examining glutaminase, the first enzyme in the glutaminolysis pathway through which the majority of aspartate in cancer cells is derived. Human GA proteins are encoded by two paralogous genes named *GLS* and *GLS2* [34], both producing several transcript variants. *GLS* gives rise to KGA and to a shorter isoform, GAC [23]. Splicing of *GLS2* leads to a long transcript named *GAB*, initially identified in human breast cancer cells, and to a shorter transcript, *LGA*, originally identified in rat liver [34,35,36]. Although the biochemical function of GA isoenzymes is the same, they appear to have opposing roles in cancer. The c-Myc-regulated *GLS* correlates with tumor growth rate and malignancy, making cancer cells dependent on glutamine anaplerosis for the maintenance of mitochondrial integrity and KC functionality [37,38]. In contrast, the role of p53-regulated *GLS2* is more controversial, with many reports supporting a tumor suppressive function [35,39,40,41,42,43,44].

For the purpose of this review, we will focus on GAC, which was initially cloned from a human colon carcinoma cell line [23]. GAC is the predominant glutaminase isoform expressed in cancer cells [26]. Furthermore, GAC and KGA display different subcellular localizations in a cell type-specific manner, mitochondrial (GAC) and cytosolic (KGA) [26]. Both isoforms are regulated by phosphate which increases their turnover rate and decreases their *K_m_*_-app_ for glutamine [26]. GAC has the lowest *K_m_*_-app_ and the highest catalytic efficiency, pointing out its central role in the increased glutaminolysis of cancer cells [26]. Glutaminase enzymatic activity in mitochondrial extracts was initially proposed in 1967 by Katunuma et al. [45], and since then, many aspects of this enzyme have been revealed: the number of encoding genes, their mechanism of splicing, expression and function regulation, kinetic constants, and inhibitors for cancer therapy. However, little has been done to shed light on the sub-mitochondrial localization of the enzyme. Early work attempted to use complementary experimental approaches to answer this question: (i) mitochondria sub-fractionation and immunoblotting with specific antisera; (ii) chemical modification with sulfhydryl group reagents of different permeability; (iii) immunological studies; (iv) enzymatic digestion at both sides of the inner mitochondrial membrane; (v) studies of intact mitochondria with [^14^C]glutamine and determination of the amount of produced [^14^C]glutamate inside and outside the mitochondria [27,28,30,46,47,48,49,50,51,52]. Unfortunately, none of these studies came to an unambiguous answer, although most studies favoured the extramitochondrial localization of the enzyme. A possible explanation for the ambiguity came from a transmission electron microscopy study of post-embedding immunogold labelling of GA in which the pig and rat renal phosphate-activated glutaminase was found partially outside and partially inside the inner mitochondrial membrane. The intermembrane space-facing GA was suggested to be the only functional enzyme, as high intramitochondrial concentrations of the inhibitor, glutamate, would keep the matrix-facing one in a dormant state [47]. The recent release of MitoCarta3.0 still reports the localization of both KGA and GAC in the IMM [53] suggesting that the true localization remains to be determined.

## 3. The Mitochondrial Glutamine Carrier: The Last Piece of Glutaminolysis Puzzle

Despite the central role of glutamine in the mitochondrial metabolism of normal and cancer cells, the gene encoding for the mitochondrial glutamine carrier remained unknown until recently. Yoo et al. [54] reported that the *SLC1A5* gene (also known as *ASCT2*) contains two different transcription initiation sites, which give rise to a long transcript encoding the plasma membrane obligatory sodium-dependent transporter for neutral amino acids [55] and to a shorter transcript (*SLC1A5_var*) encoding the mitochondrial glutamine carrier [54], although some concern about its biochemical functional has been recently raised [56]. It should be emphasized that SLC1A5_var and the mitochondrial pyruvate carrier (MPC) [57,58] do not present a tripartite structure which characterizes all members of the MCF [59,60,61]. SLC1A5 is one of the most important transporters used by cancer cells to take up glutamine [62,63]. SLC1A5 is upregulated in many forms of cancer that are characterized by rapid progression, anti-cancer drug resistance and poor survival outcome [64,65,66,67,68]. In different types of adenocarcinoma, the expression of SLC1A5_var is higher than that of surrounding normal tissue, and its expression level is correlated with poor survival. Interestingly the only reported exception is colon cancer, where outcomes are the opposite [54].

*SLC1A5_var* transcription is induced in hypoxic conditions by HIF-2α [54]. Studies of gain and loss of function demonstrated that SLC1A5_var had a key role in glutaminolysis, redox homeostasis, proliferation rate, and gemcitabine resistance of pancreatic cancer cells [54]. In pancreatic ductal adenocarcinoma (PDAC), mutated *KRAS* induces a rewiring of glutamine metabolism vital for redox homeostasis and cell proliferation [11]. In this pathway, the mitochondrial glutamine-derived aspartate once transported into the cytosol is converted through a series of enzymatic reactions to pyruvate and NADPH that cells use for reactive oxygen species (ROS) control [11,17]. Metabolomics experiments carried out with [U-^13^C]-glutamine on *SLC1A5_var* silenced PDAC cells confirmed a reduced glutaminolysis, though unfortunately the aspartate levels were not determined, although a possible reduction should be expected since a direct link between glutaminolysis and cytosolic aspartate availability in PDAC has been already demonstrated [11,17]. In fact, the proliferation defect found in *SLC1A5_var*-silenced PDAC cells could be partially restored by the addition of aspartate to the growth medium [54]. The results reported by Yoo et al. demonstrated that the GAC or its catalytic site is localized in the matrix and, at least in PDAC cells, glutaminolysis requires a mitochondrial glutamine transporter and an exporter of aspartate, identified to be UCP2 [17] (Figure 1A). Importantly, silencing of *SLC1A5_var* or *UCP2* inhibits mitochondrial glutaminolysis and abolishes gemcitabine resistance in PDAC cells [54,69,70].

## 4. The Mitochondrial Glutamate Carrier: The Other Side of the Coin

The discovery of the mitochondrial glutamine carrier pointed out that the catalytic site of glutaminase was located in the matrix. Interestingly, in colorectal cancer (CRC) the mitochondrial catabolism of glutamine required the presence of the *SLC25A22*, encoding the mitochondrial glutamate carrier isoform 1 (GC1) [71]. This suggested an external mitochondrial localization of the catalytic site of glutaminase (Figure 1B) [71].

**Figure 2:**
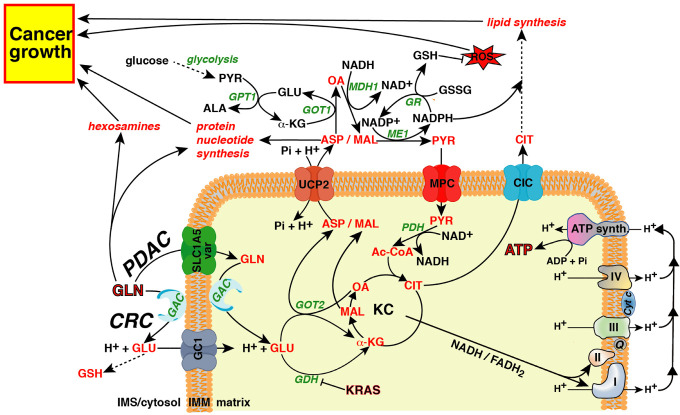
A bird’s eye view of glutamine utilization by cancer cells. Cancer cells can use the carbon skeleton and reduced nitrogen of glutamine to synthesize non-essential amino acids, hexosamines, reduced glutathione (GSH), nucleotides, proteins and lipids. Aspartate and malate produced in the matrix can be used in the cytosol for NADPH production, reducing power for the biosynthetic processes and redox homeostasis. The carbon skeleton of glutamine produces reducing equivalents in the KC (NADH and FADH_2_) which can be re-oxidized in the electron transport chain, producing chemical energy (ATP). ASP, aspartate; GLN, glutamine; GLU, glutamate, Pi, phosphate; OA, oxaloacetate; MAL, malate; α-KG, α-ketoglutarate; CIT, citrate; GSH, reduced glutathione; GSSG, oxidized glutathione; PYR, pyruvate; Ac-CoA, acetyl-CoA; ALA, alanine; Q, coenzyme Q; PDAC, pancreatic ductal adenocarcinoma; CRC, colorectal cancer; GAC, glutaminase C; SLC1A5_var, mitochondrial glutamine carrier; UCP2, uncoupling protein 2; GC1, mitochondrial glutamate carrier, isoform 1; MPC, mitochondrial pyruvate carrier; CIC, mitochondrial citrate carrier; KC, Krebs cycle; IMM, inner mitochondrial membrane; IMS, intermembrane space; GOT1/2, glutamic-oxaloacetic transaminase isoforms; GPT1, cytosolic isoform of glutamic-pyruvic transaminase; MDH1, cytosolic isoform of malic dehydrogenase; ME1, cytosolic isoform of malic enzyme; GR, glutathione reductase; PDH, pyruvic dehydrogenase; GDH, glutamic dehydrogenase.

The mitochondrial glutamate carrier in humans is encoded by two different genes *SLC25A22* and *SLC25A18,* encoding GC1 and GC2, respectively, which differ in their tissue distribution and kinetic constants [31]. Both isoforms catalyse a symport of glutamate coupled to a proton in the matrix [31]. Wong et al. demonstrated that *SLC25A22* expression increased in tumor tissues compared with non-tumor colon tissues in humans. Indeed, knockdown of *SLC25A22* in *KRAS* mutated CRC cell lines suppressed glutamine metabolism via the KC, reducing the availability of cytosolic aspartate [71,72]. *SLC25A22* silencing reduced cell proliferation, migration, and invasion in vitro, as well as tumor and metastasis formation in a xenograft model. The crucial role of the mitochondrial glutamate carrier in cytosolic availability of the glutamine-derived aspartate in *KRAS*-mutated CRC cells was further supported by the rescue of the proliferation defect induced by *SLC25A22* knockdown via the addition of aspartate in the growth medium. Most of the data reported by Wong et al. on glutamine utilization by *KRAS*-mutated CRC cell lines overlapped with those reported for the rewired glutamine metabolism induced by mutated *KRAS* in PDAC [11,17]. The only exception is that in PDAC glutamine must be transported in the matrix by the mitochondrial glutamine carrier in order to be processed by GAC, whereas in CRC this last enzymatic reaction occurs outside the mitochondria and glutamate enters into the matrix through the mitochondrial glutamate transporter (Figure 2). This difference in the glutamine utilization between PDAC and CRC may explain why although SLC1A5_var was found to be overexpressed in colon adenocarcinoma its expression was not correlated to survival outcomes [54].

Interestingly, the expression levels of GC2 in CRC tissues was lower than that of control tissues and its upregulation inhibited Warburg effect and cell proliferation via Wnt/β-catenin cascade [73]. Furthermore, high expression of GC2 indicated a longer disease-free survival time after surgery [73].

Similar to CRC, GC1 was also found to be upregulated in osteosarcoma and gallbladder cancer [74,75]. Studies of gain and loss of function showed that GC1 significantly increased osteosarcoma cell proliferation and promoted their invasion capability. Moreover, GC1 expression levels were associated with a poor outcome for patients [74]. In gallbladder cancer, the most common biliary tract malignancy, GC1 also promoted tumor development and metastasizing by activating the MAPK/ERK pathway [75].

## 5. The Mitochondrial Aspartate/Glutamate Carrier: A Key Player of Malate-Aspartate Shuttle

The first member of the MCF found to transport aspartate across the IMM was AGC [76]. In humans, there are two isoforms of AGC, AGC1, and AGC2, the former encoded by the *SLC25A12* gene, also known as *ARALAR1*, and the latter by the *SLC25A13* gene, also known as *CITRIN/ARALAR2*. AGC1 is mainly expressed in heart, skeletal muscle, and brain whereas AGC2 is expressed in many tissues and abundantly in liver [77,78]. In vitro, both isoforms catalyse a Ca^2+^-stimulated electrogenic exchange of mitochondrial aspartate for cytosolic glutamate plus a proton [76,79]. AGC, together with the mitochondrial oxoglutarate/malate carrier [80,81], the two isoforms of malate dehydrogenase and glutamic-oxaloacetic transaminase, constitute the malate-aspartate shuttle (MAS) which transfers NADH reducing equivalents from cytosol to mitochondria [82,83]. Although the key role of AGC in MAS has been demonstrated [84,85,86,87], the functionality of AGCs in certain circumstances is also crucial for cellular aspartate metabolism. In fact, AGC2 deficiency in humans causes type II citrullinemia [78,88,89], a urea cycle disease in which the low availability of cytosolic aspartate does not allow the arginine–succinate synthase to use citrulline for arginine-succinate production. Furthermore, the deficiency of AGC1 induces a global cerebral hypomyelination due to the low availability of N-acetyl-aspartate for myelin synthesis [85,90]. This means that AGC in cell metabolism may function independently of MAS, in some cases acting only to transfer aspartate out of mitochondria.

In this scenario, if GAC faces the mitochondrial intermembrane space, AGC would be enough to meet the glutamine-derived aspartate demand of cancer cells (Figure 1C). Actually, as demonstrated in a paper by Alkan et al., the knockdown of AGC1 slows down cell proliferation due to impaired aspartate synthesis [91]. Nevertheless, the total ablation of AGC1 was not sufficient to block proliferation in glutamine-replete media, suggesting the existence of at least one other pathway for the exit of aspartate from the mitochondria. Since the absence of AGC1 also sensitizes tumors to in vivo treatment with CB-839, an inhibitor of mitochondrial glutaminase, it has been proposed that this carrier sustains the growth in low glutamine conditions, and its knockdown exacerbates the growth defect observed when glutamine is limiting. To explain these observations the same authors also suggested that mitochondrial carriers that may replace AGC1 might have a higher Km for aspartate. These alternative transporters might not be sufficient to sustain aspartate export from the mitochondria when its concentration is low as seen during glutamine withdrawal [92]. This appears to be the case as the IMM contains another transporter, UCP2, able to exchange aspartate against phosphate plus a proton. The Km of UCP2 for aspartate is 6.84 mM [93], which is more than one hundred fold higher than that of AGCs (about 50 μM) [76].

A similar consideration can be made for the entry of glutamine-derived glutamate in mitochondria of CRC cells, where glutamate enters the matrix mainly through GC1 [71]; the Km of GC1 for glutamate is 5.18 mM [31] which is about thirty fold higher than that of AGCs (0.21 mM) [76]. This means that in cancer cells where glutaminolysis is very active, the high flux of metabolites across the inner mitochondrial membrane requires transporters with high Kms which are not easily saturable. Although the data reported by Alkan et al. suggest that targeting *AGC* might be an effective strategy to inhibit tumor growth in situations where nutrients are limited, other considerations must be made for alternative routes that might guarantee cytosolic aspartate availability.

## 6. Mitochondrial Uncoupling Protein 2: An Aspartate/Pi + H^+^ Exchanger Belonging to the Mitochondrial Carrier Family

UCP2 is one of the most commonly studied members of the MCF for its involvement in the cell redox homeostasis [94,95,96]. It was discovered in 1997 [97] and due to the level of amino acid identity with UCP1, the canonical uncoupler which regulates non-shivering thermogenesis in mammals, it was initially classified as an uncoupler, thus its moniker. Studies on animal models quickly ruled out its involvement in non-shivering thermogenesis and identified UCP2′s key role in redox homeostasis [94]. The first evidence of the crucial role of UCP2 in glutamine metabolism was published by Pecqueur et al. who demonstrated that UCP2 translation was under the positive control of glutamine [95]. In follow-up studies, the same research group demonstrated that *Ucp2* knock-out (KO) macrophages presented with impaired glutaminolysis [98]. *Ucp2* KO macrophages in the presence of glutamine had NADH/NAD^+^ ratios and ATP levels lower than that of their wild-type counterparts, the opposite that one would expect from an uncoupling protein which, by dissipating the electrochemical gradient across the IMM, should lower the ATP production [98]. Mitochondrial glutamine catabolism produces 4- and 5-carbon (C4 and C5) intermediates in the matrix that KC cannot fully oxidize, and these intermediates must be removed from the cycle via cataplerosis, otherwise some KC reactions would be inhibited [99].

The biochemical function of UCP2 in glutaminolysis was identified by Vozza et al. who showed that UCP2 plays a cataplerotic function, catalysing the exchange reaction of aspartate, malate, or oxaloacetate against phosphate plus a proton [93]. UCP2 is the only member of the MCF, with a cataplerotic function, able to catalyse a net efflux of aspartate out of mitochondria [100], thus making this transporter the only one suitable to work together with the mitochondrial glutamine or glutamate transporters to accomplish glutaminolysis in any type of cell (Figure 1A,B) [16]. It should be emphasized that, as reported above, the high Km of UCP2 for aspartate makes this protein unlikely to be saturated under physio-pathological conditions and to be able to guarantee large fluxes of substrates across the IMM preventing the overflow of KC due to the entry of glutamine-derived α-ketoglutarate (Figure 2).

UCP2 expression is tightly regulated at multiple levels, transcriptional, translational, and post-translational [70]. Its transcription is negatively controlled by the TGF-β signaling through SMAD4 [101], which is inactivated in over half of PDAC, and varying degrees in many other types of cancer [102,103]. At translational levels UCP2 is activated by glutamine [95,104,105] and inhibited by miRNAs, miR-133a [106] and miR-15a [107], both considered tumor suppressors and found downregulated in many types of cancer [108,109,110,111,112,113,114,115,116,117,118,119,120]. UCP2 is also post-translationally inhibited by glutathionylation [121,122], which may be considered a fine mechanism of control to regulate the cell redox-homeostasis since it has been demonstrated that increased ROS levels activate UCP2 by inducing its de-glutathionylation, and the active form of UCP2 decreases the ROS levels by increasing the GSH/GSSG ratio [17,123].

The role of UCP2 in cancer cell metabolism and chemoresistance has been demonstrated in many different cancer types [17,69,104,124,125,126,127,128,129,130,131]. The most commonly supported theory about the role of UCP2 in cancer cell proliferation and chemoresistance has been linked to its ability to reduce ROS levels by lowering the electrochemical gradient across the IMM thanks to its possible protonophoric activity. This theory was confuted by Bertholet et al. who, using a patch-clamp approach and KO mouse models, demonstrated that UCP2 was unable to catalyse fatty acid-mediated uncoupling activity [132]. Additionally, Raho et al. demonstrated that, at least in PDAC, UCP2 reduced ROS levels in *KRAS*-mutated cell lines by exporting the glutamine-derived aspartate out of mitochondria [17]. In PDAC, oncogenic KRAS induces a rewiring of the pentose phosphate pathway by decoupling the ribose 5-phosphate biogenesis from NADPH production [133]. To fulfil the NADPH needs, KRAS shifts most of the mitochondrial glutamine-derived glutamate towards GOT2 with the production of α-oxoglutarate and aspartate; the former enters in KC, and the latter, once transported to the cytosol, is converted to oxaloacetate, malate, and finally to pyruvate to produce NADPH [11] (Figure 2). In PDAC, *UCP2* silencing impairs glutaminolysis, reduces the availability of cytosolic aspartate, lowers the NADPH/NADP^+^ and GSH/GSSG ratios, and increases ROS levels [17]. Interestingly, although the inhibitory effect of *UCP2*-silencing on glutaminolysis was observed both in *KRAS*-mutated and *KRAS* wild-type cell lines, in vivo and in vitro *UCP2* silencing reduced the proliferation rate only of *KRAS*-mutated PDAC cells. These results confirmed that UCP2 is critical for glutaminolysis and the higher levels of ROS found only in *UCP2*-silenced *KRAS*-mutated PDAC cells were due to impaired glutamine oxidation, not UCP2-mediated uncoupling activity [17,134]. Of note, although UCP2 is overexpressed in many cancer types [96], its expression is not induced by *KRAS* mutations, since the expression of the G12V mutant in BxPC3, a PDAC cell line carrying the wild-type form of *KRAS*, did not alter the expression of UCP2. Interestingly, the BxPC3 cell line expressing KRAS^G12V^ showed an increased proliferation rate and clonogenic capacity which was affected by *UCP2* silencing [17], suggesting that the aspartate transport catalysed by UCP2 is crucial to support the increase of cell proliferation induced by mutated *KRAS*. Similarly to PDAC cells lacking the mitochondrial glutamine carrier, the growth defect induced by *UCP2* silencing was partially rescued by the external addition of aspartate or glutamate [17,66], suggesting that in the presence of glutamine both transporters are crucial to fulfil cytosolic aspartate needs. In contrast, when glutaminolysis is impaired or glutamine is limited, glutamate, likely through AGC [91], may enter the matrix producing aspartate which can exit with the same transporter (Figure 1C).

Most data published on the role of UCP2 in cancer comes from experiments carried out in cancer cell lines. This means that researchers have only probed the role of UCP2 in tumor maintenance and progression, whereas the role of this transporter in tumor initiation is unexplored. Recently, Aguilar et al. demonstrated that although UCP2 expression was higher in murine colorectal cancer (CRC) compared to normal tissue, its deletion enhanced colon and small intestinal tumorigenesis in carcinogen-induced and *Apc^Min/+^* mice models, respectively. This suggested a tumor-suppressive role of UCP2 in tumorigenesis [123]. During tumor initiation, the loss of UCP2 induced a metabolic rewiring in which most of the glucose-derived pyruvate was channelled towards the biosynthesis of fatty acids/phospholipids via mitochondrial synthesis of citrate. The high amounts of NADPH spent by cells for this metabolic pathway and the parallel decrease in glucose-6-phosphate dehydrogenase activity of *Ucp2* KO CRC cells reduced the GSH/GSSG ratio and increased ROS levels. The oxidative stress generated by this pathway was suggested to be the main cause of tumor initiation [123]. Although these results suggest that UCP2 has an opposite function in tumor initiation and maintenance/progression, the biochemical function of UCP2 remains the same in both situations. In fact, in CRC mice models *Ucp2* deletion impacted glucose metabolism with the loss of UCP2’s cataplerotic function increasing C4 levels in the matrix, promoting the mitochondrial utilization of the glycolysis-derived pyruvate. Similar results were also found in HepG2 and human pluripotent stem cells [93,135]. On the other hand, the UCP2-dependent export of C4 out of mitochondria impairs the pyruvate utilization in the matrix which is diverted toward lactic fermentation [93,135], and a similar mechanism of control was also exerted on fatty acid oxidation [135]. Unfortunately, the effect of *Ucp2* KO on glutamine utilization during tumor initiation in the CRC mice models was not investigated. Since the maintenance/progression of many tumors relies on glutamine utilization, the cataplerotic function of UCP2 is essential to cancer cells using glutamine as carbon and reduced nitrogen source for biosynthetic processes and redox homeostasis [11,16,17,93,98,99]. In other words, if the increase in ROS levels may be considered one of the main causes of tumor initiation, cancer cells, to survive and proliferate, quickly rewire their metabolism (Warburg effect/glutamine addiction) to control this oxidative stress. UCP2 may be considered a key metabolic switch in both processes.

## 7. Conclusions and Perspectives

A large subset of malignant tumors, in vitro and in animal models, are characterized by a glutamine addiction. As shown in Figure 2, glutamine can be used by cancer cells to synthesize most of what they need to grow and proliferate. Although in many metabolic pathways, such as nucleotide, protein, and hexosamines biosynthesis, glutamine enters directly, in others its carbon skeleton and reduced nitrogen must be reshuffled to produce other useful metabolites, such as aspartate (Figure 2, red metabolites). Aspartate can be used by cancer cells to synthesize asparagine, nucleotides, proteins and to control redox homeostasis (Figure 2).

In order to fulfil all these tasks, glutamine undergoes a series of enzymatic reactions both in mitochondria and in the cytosol, requiring continuous flux of metabolites across the IMM (Figure 2). Many mitochondrial transporters are involved in these pathways, although only those directly involved in aspartate metabolism have been covered in this review. The key role of aspartate as an endogenous metabolic limitation for tumor growth is emerging [17,21,91,92,136,137,138,139] and gain and loss of function studies have clearly demonstrated the crucial role of some of these transporters in cancer cell aspartate availability [17,54,71,72]. Many attempts aimed to target glutaminolysis of cancer cells have been focused on the design of novel and potent glutaminase inhibitors [52,140,141,142,143], some of which made it to clinical trials alone or in association with other chemotherapeutic drugs. Very little has been done to target mitochondrial transporters involved in glutaminolysis and cytosolic aspartate availability. The cause of this may be the recent discovery of their role in cancer cell metabolism [17,54,71,72] and the difficulty in carrying out inhibition assays in vitro. We believe the time has come to fill this gap. Targeting these mitochondrial transporters may provide powerful new tools to fight cancer. It should be emphasized that targeting these transporters requires a thorough understanding of the tumor-type specific metabolic signatures as the involvement of a specific transporter often depends on the type of cancer and nutrient availability (Figure 1A–C, and Figure 2). Many efforts have functionally characterized the MCF members [144] but little has been done to find specific and powerful inhibitors of the mitochondrial transporters [145]. The availability of such compounds may help fight cancer by specifically inhibiting mitochondrial metabolism as demonstrated solely for the mitochondrial citrate carrier [146,147]. An alternative strategy may be to use shRNAs or miRNAs to drastically decrease their expression levels [145]. In this context, two miRNAs, miR-15a and miR-133a known to regulate UCP2 expression, are downregulated in many kinds of cancer [70,106,107,112,113,114,115,116,117,118,148,149,150,151,152,153] and may represent an attractive therapeutic tool. CRISPR/Cas9-mediated genome editing should be considered another possible experimental approach to target these transporters by gene deletion or by knock-in of an inactivating mutation [90,100,154].

Among the four transporters considered in this review, UCP2 should be considered the most promising for three reasons:

(1) UCP2 was found to be overexpressed in many cancer types and, at least in mice, its knock-down does not produce any significant physiological alteration [97], thus it may present the advantage of obtaining drugs with minimal side effects.

(2) UCP2 would be required in both tumor types, PDAC and CRC, where glutamine and glutamate transporters are expressed (Figure 2).

(3) In tumors not dependent on *KRAS* mutation, glutamine-derived glutamate may enter in the KC through glutamate dehydrogenase, and in this case α-ketoglutarate should produce malate which can also exit from mitochondria through UCP2 [93]. Malate can be used in the cytosol to produce NADPH for redox homeostasis and reductive biosynthesis [16] (Figure 2), whereas pyruvate re-entering the mitochondria through the mitochondrial pyruvate carrier can fuel KC producing ATP or citrate for the lipid biosynthesis (Figure 2).

## Data Availability

No new data were created or analyzed in this study. Data sharing is not applicable to this article.
